# Sulfur-containing amino acids and their metabolites in atrial fibrosis

**DOI:** 10.3389/fphar.2022.1063241

**Published:** 2022-11-04

**Authors:** Rui Wang, Yong Fen Qi, Chun Hua Ding, Da Zhang

**Affiliations:** ^1^ Cardiac Department, Aerospace Center Hospital, Beijing, China; ^2^ Peking University Aerospace School of Clinical Medicine, Beijing, China; ^3^ Laboratory of Cardiovascular Bioactive Molecule, School of Basic Medical Sciences, Peking University, Beijing, China; ^4^ Key Laboratory of Molecular Cardiovascular Science, Ministry of Education, Peking University Health Science Center, Beijing, China; ^5^ Department of Pathogen Biology, School of Basic Medical Sciences, Peking University Health Science Center, Beijing, China

**Keywords:** sulfur-containing amino acid, atrial fibrosis, hydrogen sulfide, taurine, homocysteine

## Abstract

Atrial fibrosis, a symbol of atrial structural remodelling, is a complex process involved in the occurrence and maintenance of atrial fibrillation (AF). Atrial fibrosis is regulated by multiple factors. Sulfur containing amino acids and their metabolites, such as hydrogen sulfide (H_2_S) and taurine, can inhibit the process of atrial fibrosis and alleviate atrial remodeling. However, homocysteine can promote the activation of atrial fibroblasts and further promote atrial fibrosis. In this review, we will focus on the recent progress in atrial structural changes and molecular mechanisms of atrial fibrosis, as well as the regulatory roles and possible mechanisms of sulfur containing amino acids and their metabolites in atrial fibrosis. It is expected to provide new ideas for clarifying the mechanism of atrial fibrosis and finding targets to inhibit the progress of atrial fibrosis.

## Introduction

It is estimated that the prevalence of atrial fibrillation (AF) in adults is between 2% and 4%, and a 2.3-fold rise is expected (Hindricks et al., 2021). As a major risk factor of cardiovascular events, stroke and sudden death, AF brings heavy economic and medical burden to individuals and society (Christophersen et al., 2017). Atrial fibrosis is the main pathophysiological factor for the occurrence and maintenance of AF (Sohns and Marrouche, 2020). Clinical studies indicate that AF is mainly due to atrial structural remodeling (van den Berg et al., 2021), atrial structural remodeling have been found even before the occurrence of AF in patients. Inhibition of atrial structural remodeling caused by atrial fibrosis is the key direction to inhibit the occurrence and further development of AF. Exploring the mechanisms and intervention strategies of atrial fibrosis can delay the progress of fibrosis, and then reduce the structural and electrical remodeling of the atrium, playing a pivotal role in the treatment of AF.

Many kinds of sulfur-containing amino acids and their metabolites are responsible for cardiovascular disease. Changed plasma sulfur-amino acid level are found in cardiovascular diseases, indicating the importance of proper regulation of thio metabolism for cardiovascular homeostasis (Mistry and Brewer, 2019). For example, H_2_S, the sulf-metabolite of methionine, plays a biological protective role such as reducing extravascular collagen deposition, inhibiting cardiovascular inflammatory reaction, relaxing blood vessels, attenuating cardiomyocyte apoptosis and inhibiting vascular smooth muscle cell proliferation (Zhang et al., 2019). In recent years, there is growing focus on the role of sulfur-containing amino acids and their metabolites in anti-atrial fibrosis. In this review, we will discuss the formation of atrial fibrosis, the metabolism of sulfur-containing amino acids and their role in the formation and progression of atrial fibrosis, and provided new insights into potential clinical applications and interventions.

## Structural changes and molecular mechanisms of atrial fibrosis

**FIGURE 1:**
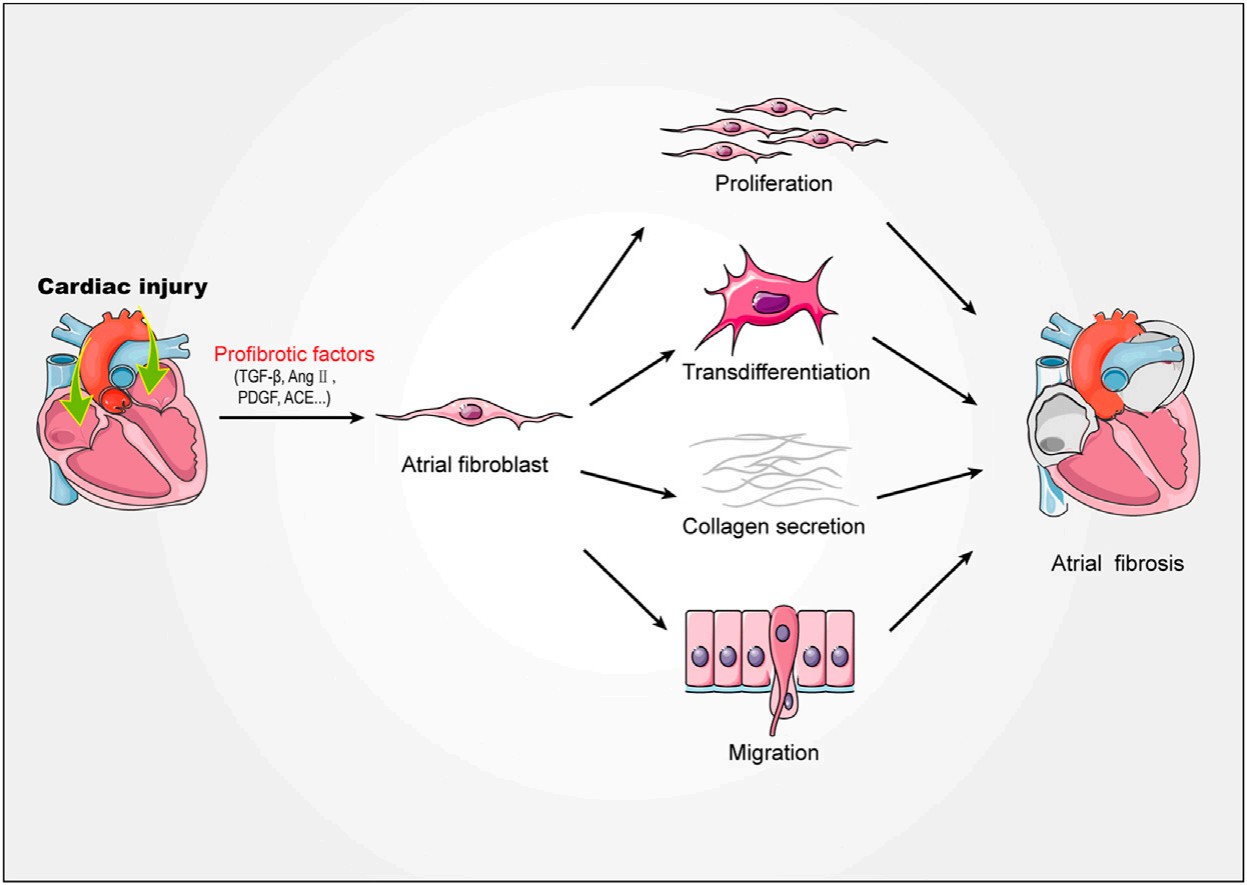
Mechanism of atrial fibrosis. Stimulated by injury factors TGF-β, AngⅡ, PDGF, ACE, etc. Atrial fibroblasts proliferate, migrate, transdifferentiate, secrete collagen fibers, and ultimately promote the formation of atrial fibrosis. TGF-β, transforming growth factor-β; AngⅡ, angiotensin II; PDGF, platelet derived growth factor; ACE, angiotensin-converting enzyme.

Atrial fibroblasts are the major effector cells of atrial fibrosis, which would be activated, proliferated and transdifferentiated into myofibroblast during fibrosis (Krishnan et al., 2021; Li et al., 2021). The myofibroblasts showed greater mobile and contractile and produced a large amount of extracellular matrix (Frangogiannis, 2019, 2021). Under physiological conditions, atrial resident fibroblasts would rest and produce less extracellular matrix (Souders et al., 2009). However, when atrium was stimulated by mechanical injury, pressure overload, electrical stimulation and other stimulators, extracellular matrix would be abnormally deposited in tissues, leading to atrial fibrosis. The extracellular matrix is mainly composed of collagen I and collagen III (Li et al., 2018). Collagen I is the main structural component of cardiac interstitium, which is similar to fibers and mainly forms thick and hard rod-shaped structures around the outer membrane and muscle. In contrast, collagen III forms thinner and more flexible reticular fibers, which is more abundant in endocardium, thus increasing the elasticity of heart tissue (Frangogiannis, 2019). When atrial fibrosis was activated, the synthesis/degradation of atrial collagen I and collagen III proteins changed seriously, which lead to atrial remodeling. This pathophysiological process, which characterized by abnormal collagen metabolism, is related to the disorder of matrix metalloproteinase/tissue metalloproteinase inhibitor (MMP/TIMP) system and the increase of Kazal motif (RECK) level. In addition, previous studies showed that the expression of non fibrous collagen VI was up-regulated in patients with AF during the process (Polyakova et al., 2008). Although non fibrous collagen did not form large fiber bundles, they could still interact with collagen I and collagen III, which promoted the process of atrial fibrosis (Polyakova et al., 2008) (Figure 1).

Atrial fibrosis was divided into reactive fibrosis and repair fibrosis (Krishnan et al., 2021; S. Nattel, 2017). Atrial reactive fibrosis was considered a response fibrous tissue to cardiac inflammation or pressure overload. When the atrium initiated reactive fibrosis, the thin-fibrous tissue which layer around the myocardial bundle changed into thick sheath. By isolating muscle bundles, myocardial interstitial fibrosis could accelerate conduction. The presence of thick interstitial collagen strands could be associated with more rapid longitudinal conduction and persistent AF in patients (Krul et al., 2015). When the damage or death of myocardial caused by various factors occured, the repair fibrosis would be initiated, such as myocardial infarction (MI) scar. This type of fibrosis might be irreversible to a large extent. Different types of fibrosis would not necessarily mutually exclusive, and patients with atrial remodeling were likely to combine reactive fibrosis and repair fibrosis (Nattel et al., 2017).

Atrial remodeling associated with atrial fibrosis which could be divided into structural remodeling and electrophysiological remodeling (Pellman et al., 2010). When the atrial fibrosis process get started, the structural and electrical remodeling will be activated, such as inducing left atrial dysfunction (LADS) and increasing susceptibility to AF (Hu et al., 2021). Clinical studies indicated that the persistence AF was mainly attributed to structural remodeling (Yang et al., 2017; Beyer et al., 2021). In fact, cardiac fibroblasts are non excitable cells essentially, but they can transfer electric current between cardiac fibroblasts and myocardial cells through connexin (Andrade et al., 2014). The continuous accumulation of fibrin would lead to permanent tissue remodeling and organ damage. In the atrium, the continuous accumulation of fibrin will directly affect the mechanical function and electrical activity of the myocardium, leading to abnormal cardiac systolic and diastolic functions. These pathological processes change the electrophysiological characteristics of the original myocardium, leading to abnormal atrial electrical conduction and reentry of the atrial loop, thus directly or indirectly leading to arrhythmia, and thus leading to the occurrence and progress of AF (Pellman et al., 2010; Afzal et al., 2017). Therefore, prevention of atrial remodeling which related to atrial fibrosis is the key to prevent AF.

At present, several common mechanisms of promoting fibrosis have been identified. The classic Smad dependent pathway and non classic Smad independent pathway activated downstream of transforming growth factor (TGF-β) (Biernacka et al., 2011; Saadat et al., 2020), the activation of renin angiotensin aldosterone system (RAAS) (AlQudah et al., 2020), oxidative stress, and the activation of PDGF-JAK-STAT pathway mediated by platelet derived growth factor (PDGF) have been shown to be closely related to cardiac fibrosis (Everett and Olgin, 2007; Li et al., 2021). By reviewing the literature, we found that under different pathophysiological conditions, atrial and ventricular might exhibit different levels of fibrosis (Nakajima et al., 2000). Transgenic mice with TGF-β1 exhibited higher TGF-β1 levels in the atria than in the ventricles under the control of an α-MHC promoter. In this model, as shown by RNA microarray analysis, 80 fibrogenic genes are overexpressed in atrium, while only 2 genes are differentially expressed in ventricle (Rahmutula et al., 2013). Similarly, transgenic mice overexpressing angiotensin-converting enzyme (ACE) showed atrial hypertrophy and dilation with focal atrial fibrosis, but the ventricles were normal (Xiao et al., 2004). This specific fibrotic response to ACE overexpression in different parts of the heart could be partly explained by the different expression of AT1 receptor in atria and ventricles (Bauer et al., 1997). Studies have shown that atrial fibroblasts are stronger than ventricular fibroblasts in terms of TGF-β1 mediated fibrosis and oxidative response (Yeh et al., 2013). TGF-β1 through TGF-β1/Smad pathway phosphorylates Smad 2 to promote atrial fibrosis. These processes eventually lead to the accumulation of fibrous and non fibrous collagen, which in turn leads to excessive atrial fibrosis and AF (Polyakova et al., 2008). This indicates that the atrium has a stronger fibrotic response to various stimuli (Burstein et al., 2008). The results above indicate that the mechanisms of fibrosis in atrial and ventricular are different. Compared with ventricles, further studies are needed to determine whether other special signal pathways or mechanisms contribute to the development of atrial selective fibrosis.

## The mutual transformation mechanism of sulfur-containing amino acids and their metabolites

Methionine (Met), as the starting amino acid for protein synthesis in eukaryotes, is one of the common sulfur containing amino acids. Met metabolism includes transmethylation, demethylation and transvulcanization.

In the process of methylation, Met is activated by methionine adenosyltransferase (MAT) to S-adenosylmethionine, and then S-adenosylmethionine transfers S-adenosylmethionine methyl through methyltransferase (MT) to S-adenosylhomocysteine. Finally, S-adenosylhomocysteine is hydrolyzed into adenosine and homocysteine (Hcy) by S-adenosylhomocysteine hydrolase (SAHH) to complete transmethylation (Das et al., 2019; Elwan et al., 2021). The demethylation of Hcy to Met is called demethylation. Under the catalysis of methionine synthetase (MTR), with vitamin B12 as the coenzyme, 5-methyltetrahydrofolate as the methyl donor, Hcy obtains methyl to generate Met (Portillo et al., 2020).

The catabolism of Met is mainly through transvulcanization. After Met is converted into Hcy, Hcy and serine (Ser) are condensed to form cystathionine under the catalysis of cystathionine β-synthase (CBS) (Hage et al., 2021). Next, cystathionide is catalyzed by cystathionine γ-lyase (CSE) to produce cysteine. Then, with cysteine as the central link, cysteine generates thiometabolic products through multiple metabolic pathways, which is called transvulcanization (Townsend et al., 2004).

There are several further catabolic pathways of cysteine generated in the process of sulfurization. First of all, cysteine can form cystathionine and H_2_S with Hcy under the catalysis of CSE. Cysteine transvulcanization is further cracked into cysteine (Cys), α-Ketobutyrate and ammonia under the catalysis of CSE, which are involved in metabolism, such as entering the tricarboxylic acid cycle or excreted by urine (Portillo et al., 2020). Secondly, Cys can be further decomposed into cystine, which is catalyzed by CSE to produce thiocysteine and pyruvate. Thiocysteine and pyruvate are further decomposed to produce cysteine and H_2_S. At the same time, cysteine can also generate 3-mercaptopyruvate under the action of 2-cysteine aminotransferase (CAT), and 3-mercaptopyruvate can generate H_2_S and pyruvate under the further catalysis of 3-mercaptopyruvate sulfurtransfer (3-MST), which is carried out in mitochondria (Kolluru et al., 2022).

**FIGURE 2:**
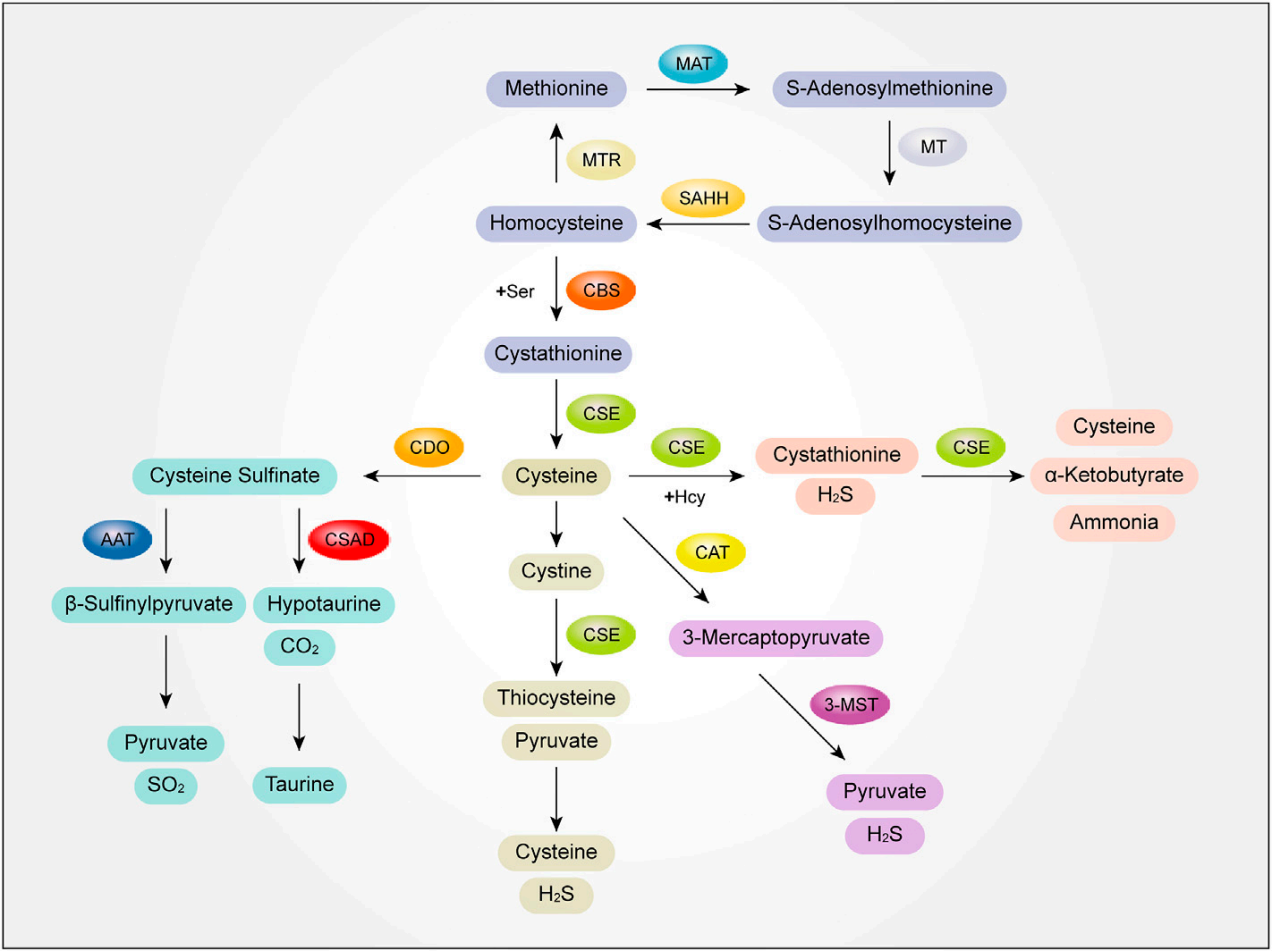
The mutual transformation mechanism of sulfur-containing amino acids and their metabolites. MAT, methionine adenosyltransferase; MT, methyltransferase; SAHH, S-adenosylhomocysteine hydrolase; MTR, methionine synthetase; Ser,serine; CBS, cystathionine β-synthase; CSE, cystathionine γ-lyase; Hcy, homocysteine; H_2_S, hydrogen sulfide; CAT, 2-cysteine aminotransferase; 3-MST, 3-mercaptopyruvate sulfurtransfer; AAT, aspartate aminotransferase; CSAD, cysteine sulfite decarboxylase; SO_2_, sulfur dioxide.

In addition, the catabolism of cysteine can also produce sulfur dioxide and taurine. Cys is first oxidized to form cysteine sulfinate under the action of cysteine dioxygenase (CDO). Cysteine sulfinate generates β-sulfinylpyruvate through transamination under the catalysis of aspartate aminotransferase (AAT). Then β-sulfinylpyruvate decomposes spontaneously into pyruvate and sulfur dioxide. Cysteine sulfinate can also generate sub taurine and carbon dioxide under the action of cysteine sulfite decarboxylase (CSAD), and then generate taurine (Huang et al., 2016) **(** Figure 2).

## Mechanism of sulfur-containing amino acids and their metabolites in atrial fibrosis

### Homocysteine and atrial fibrosis

Homocysteine (Hcy) is a non essential amino acid containing sulfur. As an independent risk factor of cardiovascular disease, Hcy plays a complex role in the pathophysiology of cardiac remodeling (Yao et al., 2017). Cardiovascular diseases with hyperhomocysteinemia (hHcy) are significantly related to cardiac fibrosis (Kar et al., 2019; Deng et al., 2022). Clinical studies have found that hHcy can promote cardiac hypertrophy in patients with hypertension. In addition, Hcy can promote cardiac inflammation and fibrosis by exacerbating AngII-stimulated expression of Calcineurin and nuclear factor of activated T cells (NFAT) (Deng et al., 2022).

Atrial fibrosis is an inevitable pathological determinent of several different heart diseases, especially AF. High plasma Hcy is considered to be a risk factor for AF (Nasso et al., 2013; Yao et al., 2017) and is closely related to the formation of atrial fibrosis (Svenningsson et al., 2020). Atrial structural remodeling can be detected in patients with both paroxysmal or permanent AF. HHcy is an key pathological feature in the etiology of atrial fibrosis (Iguchi et al., 2018). The activation level of transient receptor potential 3 (TRPC3) channel is closely related to the degree of atrial fibrosis and the incidence rate of AF. It was found that Hcy mediates atrial fibrosis by triggering the activation of TRPC3 signaling pathway (Han et al., 2020). TRPC3 is an indispensable factor in regulating the development mechanism of fibrosis and promoting the transformation of fibroblasts into myofibroblasts, which has adverse effects on the regulation of collagen (Numaga-Tomita et al., 2017). Hcy can combine with G protein coupled receptor (GPCR) and activate phospholipase C (PLC) to produce intracellular messenger 1,2-diacylglycerol (DAG). DAG recruits protein kinase C (PKC) and subsequently affects the downstream signaling pathway of cardiac fibrosis (Vacek et al., 2009). TRPC3 is directly activated by PKC phosphorylation. Higher Hcy level can combine with GPCRs, thus activating the downstream PKC/TRPC3 signal pathway, promoting the formation of atrial fibrosis. On the other hand, studies have found that atrial fibrosis induced by hHcy is also mediated by the interaction between TRPC3 channel and sirtuin-1 (SIRT1). Patients with hHcy showed elevated TRPC3 protein levels, decreased SIRT1 levels, and activation of TGF-β signal pathway. In addition, the experiment showed that TAC mice fed with hHcy developed heart failure which the type of preserved ejection fraction (HFpEF) accompanied with atrial fibrosis. Over-expression of SIRT1 and knockdown of TRPC3 could effectively control Hcy mediated atrial fibrosis in mice. The results showed that TRPC3 is required for the activation and translocation of TGF-β into the nucleus of fibroblasts by mediating the trafficking and activity of SIRT1. The clinical analysis also showed that compared with the sinus rhythm group, the level of TRPC3 RNA in patients with AF increased, while the level of SIRT1 RNA decreased.

In conclusion, the increase of Hcy level promotes the proliferation and differentiation of atrial fibroblasts, which shows that Hcy can combine with GPCRs and increase the protein level of TPRC3. TRPC3 is a negative regulator of SIRT1. Under the stimulation of homocysteine, TRPC3 can directly interact with SIRT1, leading to activation of TGF signal pathway, thereby inducing collagen accumulation and forming atrial fibrosis (Han et al., 2020). But the molecular interaction between Hcy and atrial myocytes and atrial fibroblasts remains to be clarified.

### Taurine and atrial fibrosis

Taurine is a conditionally essential amino acid, which is expressed in most animal tissues. In recent years, research has found that taurine, with its characteristics of anti-oxidative stress (Baliou et al., 2021) and anti-inflammation (Qaradakhi et al., 2020), plays a protective role in cardiovascular diseases such as reducing atherosclerosis, improving myocardial function and reducing cardiovascular risk factors (Zulli, 2011). Taurine can reduce myocyte hypertrophy and fibrosis by activating SIRT1-p53 pathway, significantly relieving cardiac dysfunction after TAC (Liu et al., 2020). Futher more, taurine can reduce iron mediated cardiac fibrosis by playing the role of anti-oxidative stress (Oudit et al., 2004). The lack of taurine can cause atrophic cardiac remodeling in wistar rats (Pansani et al., 2012). It is also found that the anti-atrial structural remodeling effect of taurine is related to its anti-inflammatory effect (Yang et al., 2017). In the rats with atrial fibrosis, taurine significantly decreased the serum or plasma levels of tumor necrosis factor-α (TNF-α), interleukin-6 (IL-6), AngII, high-sensitive C-reactive protein (Hs-CRP) and matrix metallaproteinase-9 (MMP-9). Meanwhile, it reduced the degree of interstitial fibrosis significantly. Electron microscopy showed that taurine could significantly reduce the ultrastructure damage of atrial cells, and could reverse the change of gap junction and maintain the integrity of myocardial ultrastructure. The results indicated that taurine could reduce the degree of atrial fibrosis partly by reducing the levels of inflammatory factors and fibrogenic molecules. Taurine can inhibit atrial structural remodeling caused by AF through by protecting the integrity of myocardial ultrastructure (Yang et al., 2017). However, the potential mechanism of taurine regulating the activation of inflammatory signaling pathway and inhibiting atrial fibrosis still needs to be further explored.

### Hydrogen sulfide and atrial fibrosis

At present, hydrogen sulfide (H_2_S) has been defined as the third gas signal molecule after nitric oxide (NO) and carbon monoxide (CO) (Zhao et al., 2022). *In vivo*, endogenous H_2_S is mainly produced by CBS, CSE and (3-mercaptosulfurtransferase) 3-MST, among which 3-MST is the key enzyme in mitochondria to produce H_2_S (Bełtowski, 2015). In the cardiovascular system, H_2_S is mainly generated endogenously through the catalysis of CSE and participates in a variety of physiological and pathophysiological processes such as inhibition of oxidative stress, inflammation, cell death, cell proliferation and transformation (Zhang et al., 2019). Clinical studies have found that the expression of CSE and 3-MST in the left atrial appendage of patients with AF is decreased, and the expression of CBS is increased, accompanied by significant atrial fibrosis. Patients with AF showed that the significantly reduced level of H_2_S in plasma (Hu et al., 2021). Further experiments showed that these changes were related to the increased warburg effect and the occurrence of endoplasmic reticulum stress (ERS). Supplementation of H_2_S can reduce atrial fibrosis by inhibiting warburg effect and ERS induced by AngII (Hu et al., 2021). Another study confirmed that during the occurrence of AF, stable sulfide stored in mitochondria was mobilized to produce free sulfide in the blood to offset myocardial damage caused by oxidative stress (Watts et al., 2021). Therefore, the longer the duration of AF, the long-term stable sulfide storage reserves are consumed more, which leads to the decreased plasma H_2_S content and the intensification of oxidative stress reaction, thus aggravating the occurrence of atrial fibrosis (Watts et al., 2021). Compared with the atrial tissues of patients with sinus rhythm, the expression of CSE in atrial tissues of AF was lower and atrial fibrosis was significant. The level of CBS in patients with AF is significantly higher than that in patients with sinus rhythm, which may be related to feedback regulation of CSE and CBS (Nandi and Mishra, 2017).

As an endogenous gas transmitter, H_2_S also plays an pivotal role in many cell systems by regulating the activity of ion channels. H_2_S was the first opener of (ATP Sensitive K^+^) KATP channel identified in vascular smooth muscle cells (Tang et al., 2005). Through activation of KATP channels, H_2_S lowers blood pressure, protects heart from ischaemia and reperfusion injury (Givvimani et al., 2011; J. Huang et al., 2012). Interestingly, other studies have found that the mechanism of H_2_S inhibiting atrial fibrosis is independent of KATP channel. H_2_S can potentially regulate cardiac fibrosis by inhibiting the activation of large conductance Ca^2+^-activated K^+^current (BKCa), transient outward K^+^current (IK_to_) and inward rectifying K^+^current (IK_ir_), leading to decreased proliferation and suppression of transforming growth factor-β1 (TGF-β1)–induced myofibroblast transformation of atrial fibroblasts (Sheng et al., 2013). In addition to the ion channel pathway, H_2_S can also reduce the incidence rate of AF and alleviate atrial fibrosis by activating PI3K/Akt/eNOS pathway (Xue et al., 2020). Su et al. (2021). found in the model of fibrosis in human atrial fibroblasts (HAFs) induced by Ang II that the proliferation and migration of HAFs were enhanced, the expression of microRNA-133a (miR-133a) was reduced, and connective tissue growth factor (CTGF) and fibrosis markers such as collagen I, collagen III and the level of α- SMA increased. After H_2_S intervention, the fibrosis, proliferation and migration of HAFs induced by Ang II were reduced. Further research found that CTGF was the direct target of miR-133a to regulate atrial fibrosis. H_2_S improved HAFs fibrosis by significantly increasing the expression of miR-133a.

## Summary and prospect

Atrial fibrillation (AF) is the most common malignant arrhythmia in clinic. It is estimated that more than 33 million people worldwide are affected by AF (Christophersen et al., 2017). In recent years, China has become the country with the largest number of patients with AF in the world, and the prevalence of AF will further increase with the aging of the population in China (Christophersen et al., 2017; Hindricks et al., 2021). Currently, research shows that atrial fibrosis is an important reason for the occurrence and progress of AF, and its formation and progress are related to transforming growth factor1 (TGF-β1), renin angiotensin aldosterone system (RAAS), inflammation, rlatelet-derived growth factor (PDGF) and miRNAs (Li et al., 2021). However, the mechanisms have not been fully clarified. More and more studies have focused on the important role of sulfur-containing amino acids and their metabolites in the formation and development of atrial fibrosis. Taurine and H_2_S inhibit atrial fibrosis through their anti-inflammatory effects and ion channel regulation (Sheng et al., 2013; Yang et al., 2017), and homocysteine inhibits atrial fibrosis by promoting the interaction between TRPC3 and SIRT1 (Han et al., 2020). These findings provide new ideas for the prevention and treatment of atrial fibrosis and AF.

Although people have known some about the operations of sulfur-containing amino acids and their metabolites (Townsend et al., 2004; Kolluru et al., 2022), there are still a lot of specific mechanisms, mediators and conditions which might affect the progression of atrial fibrosis need to be explored. For example, how the metabolites of sulfur-containing amino acids affect the progression of atrial fibrosis?How the key enzymes of sulfide synthesis play a role in atrial structural remodeling and electrical remodeling?What are the direct targets of sulfur-containing amino acids and their metabolites in promoting or inhibiting the formation and progression of atrial fibrosis? The role of sulfur-containing amino acids and their metabolites in the occurrence and development of atrial fibrosis and the related molecular mechanisms have not been clarified, and a lot of in-depth research works are still needed in the future. Further elucidating the mechanism of sulfur-containing amino acids and their metabolites in the development of atrial fibrosis will provide a new basis for early diagnosis, prevention and targeted treatment of atrial fibrosis.
